# Chloride of Zinc

**Published:** 1859-07

**Authors:** J. Taft


### CHLORIDE OF ZINC.

BY J. TAFT.

IN the hands of the surgeon, this is one of the most powerful and efficient escharotics. In the hands of the dental surgeon, when properly employed, it is equally efficient; with it, results are obtained more promptly and efficiently than with any other agent. It is used by the dentist chiefly for destroying exalted sensibility of the dentine. In the great majority of cases, it destroys the sensitiveness of a considerable lamina of the diseased portion, in a very few moments.

While this is being accomplished, by the pure crystal chloride, a sharp, pungent pain is experienced by the patient, which, in highly sensitive constitutions, is almost intolerable; and this prevents many operators from using it at all. Various means have been employed to obviate this difficulty,—such as diluting with flour, and plaster of paris, or applying with it chloroform or creosote, or something of this character. All these, except chloroform, are in some respects objectionable.

There is an article in the May number of the London *Lancet*, by J. C. Wardsworth, in which he recommends diluting the chloride with oxyd of zinc: he recommends this preparation for surgical use.

I have prepared it, and been using it, as an application for sensitive dentine, with very good effect. Its action is less rapid but quite as efficient.

The oxyd of zinc is a very proper material with which to dilute the chloride. When applied to sensitive dentine, it produces far less pain than the chloride alone, though longer time is required to produce its specific result.

It is prepared by mixing and triturating equal proportions of the dry chloride and the oxyd of zinc.

"The mixture should be preserved in a wide-mouthed bottle, having an accurately-fitting stopper; it is said a chemical combination occurs between the chloride and oxyd, but, so far as can be decided, no great loss of activity will result." It is difficult to keep the pure chloride from deliquescence. When exposed to the air, it contracts water very rapidly, and by this means, parts with much of its chlorine, and so loses its efficient principle.

When pulverized with the oxyd, however, this difficulty is obviated; for the mixture remains a perfectly dry powder.

The pure crystal may be kept perfectly free from change in chloroform.

### CASE OF SENSITIVE DENTINE.

BY J. TAFT.

